# Ethical considerations of prospective data collection for stereotactic arrhythmia radioablation (STAR): an overview from the STOPSTORM.eu consortium

**DOI:** 10.1007/s00066-025-02480-2

**Published:** 2025-10-29

**Authors:** Chiara Crico, Oliver Blanck, Marcin Miszczyk, Melanie Grehn, Stefano Mandija, Luis Schiappacasse, Jakub Cvek, Tomasz Jadczyk, Steen Buus Kristiansen, Manuel Algara López, Gaetano Maria De Ferrari, Pieter G Postema, Martin F Fast, Etienne Pruvot, Joost J C Verhoeff, Nicolaus Andratschke, Slawomir Blamek, Marta Perin, Ludovica De Panfilis

**Affiliations:** 1 Legal Medicine and Bioethics, AUSL-IRCCS Reggio Emilia Hospital, Italy—Fondazione IRCCS Istituto Tumori di Milano, Milan, Italy; 2 Department of Radiation Oncology, University Medical Center Schleswig-Holstein, Kiel, Germany; 3 Department of Urology, Comprehensive Cancer Center, Medical University of Vienna, Vienna, Austria; 4 Collegium Medicum—Faculty of Medicine, WSB University, Dąbrowa Górnicza, Poland; 5 Department of Radiotherapy, University Medical Center Utrecht, Utrecht, The Netherlands; 6 Computational Imaging group for MR therapy and diagnostics, Division Imaging and Oncology, University Medical Center Utrecht, Utrecht, The Netherlands; 7 Department of Oncology, Service of Radiation Oncology, Lausanne University Hospital and University of Lausanne, Lausanne, Switzerland; 8 Department of Oncology, University Hospital and Faculty of Medicine Ostrava, Ostrava, Czech Republic; 9 3rd Department of Cardiology and Structural Heart Diseases, Medical University of Silesia, Katowice, Poland; 10 Faculty of Space Technologies, AGH University of Krakow, Krakow, Poland; 11 Interventional Cardiac Electrophysiology Group, International Clinical Research Center, St. Anne’s University Hospital in Brno, Brno, Czech Republic; 12 Heart Rhythm Centre, Royal Brompton and Harefield Hospitals, Guy’s and St Thomas’ NHS Foundation Trust, Hill End Road, Harefield, London, UK; 13 School of Biomedical Engineering and Imaging Sciences, Faculty of Life Sciences and Medicine, King’s College London, London, UK; 14 Willem Einthoven Center for Cardiac Arrhythmia Research and Management, Leiden, The Netherlands; 15 Willem Einthoven Center for Cardiac Arrhythmia Research and Management, Aarhus, Denmark; 16 Department of Cardiology, Aarhus University Hospital, Aarhus, Denmark; 17 Department of Radiation Oncology, Hospital del Mar, Hospital del Mar Research Institute, Departament de Medicina i Ciències de la Vida, Universitat Pompeu Fabra, Barcelona, Spain; 18 Division of Cardiology, Department Cardiovascular and Thoracic, Città della Salute e della Scienza, University of Turin, Turin, Italy; 19 Heart Center, Department of clinical and experimental cardiology, Heart Failure & Arrhythmias, Amsterdam Cardiovascular Science, Amsterdam University Medical Centers, University of Amsterdam, Amsterdam, The Netherlands; 20 Heart and Vessel Department, Service of Cardiology, Lausanne University Hospital and University of Lausanne, Lausanne, Switzerland; 21 Department of Radiotherapy, Amsterdam University Medical Centers, Amsterdam, The Netherlands; 22 Department of Radiation Oncology, University Hospital of Zurich, The University of Zurich, Zurich, Switzerland; 23 Department of Radiotherapy, Maria Skłodowska-Curie National Research Institute of Oncology, Gliwice, Poland; 24 Department of Medical and Surgical Sciences, University of Bologna, Azienda Ospedaliero-Universitaria IRCCS Bologna, Bologna, Italy; 25 Fondazione IRCCS Istituto dei Tumori di Milano, Milano, Italy

**Keywords:** Ventricular Tachycardia, Research Ethics, Stereotactic Body Radiotherapy SBRT, Clinical Trials, Ethical and Regulatory Challenges

## Abstract

**Background:**

Ventricular tachycardia (VT) is a life-threatening condition, and standard treatments are not suitable for many affected patients. Stereotactic arrhythmia radioablation (STAR) has emerged as a promising experimental last-resort treatment for patients with refractory VT, but it lacks clinical standardization. To address this issue, the STOPSTORM.eu consortium aims to collect data on patients treated with STAR via the development of a multicentric patient registry. The Ethics & Regulations Working Group (ERG) provides support addressing ethical and regulatory challenges.

**Methods:**

The ERG conducted a survey to assess how prospective data on STAR are collected at the partner centres and to explore potential ethical concerns. Responses were analysed to evaluate clinical trial approval processes, adherence to STOPSTORM guidelines, and emerging ethical issues.

**Results:**

Among the 28 partners, there were 13 interventional clinical trials—ongoing or concluded—across seven countries; centres without ongoing trials enrolled patients under compassionate use. Most trials were single arm, with few exceptions (a randomized trial and dose escalation studies). Most ethics committees approved STAR trials without major objections, but regulatory inconsistencies were observed, resulting in approval denial or delay. Ethical concerns included potential therapeutic misconception among patients, autonomy issues due to the vulnerability of VT patients, and inequities in access to STAR. The heterogeneity in trial designs, endpoints, and follow-up strategies among participating centres posed challenges for data standardization, but using a registry to collect data from multiple local clinical trials offers an innovative approach to overcoming logistical and financial barriers in research on rare diseases.

**Conclusion:**

Multicentric non-pharmaceutical trials on STAR may present ethical, regulatory, and organizational challenges. The open registry model facilitates large-scale data collection and supports future protocol standardization. However, greater intercentre collaboration and regulatory harmonization are needed to optimize STAR’s integration into clinical practice while upholding ethical standards in patient care and research.

**Supplementary Information:**

The online version of this article (10.1007/s00066-025-02480-2) contains supplementary material, which is available to authorized users.

## Background

Ventricular tachycardia (VT), a complex arrhythmia of ventricular origin, is an unpredictable and life-threatening condition [1]. Patients with VT are conventionally treated with antiarrhythmic medication and receive an implantable cardioverter defibrillator (ICD) that interrupts symptoms and reduces the risk of sudden cardiac death. The underlying cause of the disease, the VT substrate, can be treated locally with catheter ablation (CA). Unfortunately, clinicians are often unable to treat the entire substrate, which, combined with progression of the underlying cardiac disease, leads to VT recurrences in 20–50% of patients [2]. This exposes the patients to iterative hospitalization and repetitive painful, frightening, or life-threatening ICD interventions that have a strong impact on the psychological wellbeing of patients and negatively affect their quality of life (QoL) [3]. It is often possible to perform repeat CA; however, further recurrences are common, and a subset of patients is eventually left without effective therapy. Stereotactic arrhythmia radioablation (STAR), also referred to as cardiac stereotactic body radiotherapy (SBRT), was developed as a bail-out therapy in these patients [4, 5].

To date, ten single-arm prospective trials on STAR have published their interim or final results, showing consistently high response rates with regard to VT burden reduction as well as moderate toxicity, with approximately one in ten patients experiencing a severe or worse treatment-related adverse event; however, at the same time, VT recurrences remain common. By 1 year, as many as four in five patients experience VT recurrence or pass away [6]. Still, the reduction in VT burden due to STAR, often also combined with a reduction in anti-arrhythmic medication, putatively associates with the often-observed improvement in quality of life [3]. Due to the low volume of data and heterogeneity between centres, the European Union Horizon 2020-funded Standardized Treatment and Outcome Platform for Stereotactic Therapy of Re-Entrant Tachycardia by a Multidisciplinary (STOPSTORM) Consortium was initiated to collect data on the use of STAR in VT patients, with the main goals of standardization and determination of the clinical outcomes of STAR through the development of a multicentric patient registry comprising both prospectively and retrospectively recruited cohorts of patients [7].

Patients with refractory VT have very limited standard options; however, since STAR remains experimental, it is currently regarded as a last-resort or bail-out treatment. Consequently, these patients are at a higher risk of exploitation and should thus be considered as vulnerable subjects [8]. This requires special care and caution, which is why the prospective patient recruitment in STOPSTORM is paralleled by a dedicated research group providing ethical guidance and regulatory support (the Ethics & Regulations Working Group or ERG^1^). The aim of the ERG is to define the ethical and legal framework for STAR [7; 8] and to conduct empirical research on the ethical dimensions involved in the project. In this study, we aimed to explore the ethical, regulatory, and organizational challenges associated with the use of STAR within the STOPSTORM.eu consortium. Using a questionnaire, we investigated how prospective data are collected among participating centres, how local ethics committees interpret and evaluate STAR protocols, and which ethical concerns potentially emerge from STAR treatment practices. The study also reflects on the opportunities and limitations of using an open registry model to support clinical research on non-standardized non-pharmaceutical interventions based on the survey results and using the STOPSTORM experience as a case study.

## Methods

Initially, the ERG reviewed and investigated the ethically relevant aspects concerning the use of STAR: patient recruitment process (via clinical trial and/or compassionate use of STAR), study design (if any), and the approval processes for the research protocols. The ERG partners developed a survey investigating these topics and distributed it to the STOPSTORM national coordinators (NC) in March 2022. The NCs were responsible for delivering the survey to each national STOPSTORM partner site. Reminders were sent in April and May 2022.

Following initial responses, the survey was revised based on feedback from the participating centres. The updated version was circulated among the NCs and the STOPSTORM members in June 2022. In October 2022, the ERG added questions to gather more details on the clinical trial submission process and track each partner’s progress (see Appendix 1 for all survey versions), and the final survey was sent out with a final reminder. Data collection was completed in November 2022. For two centres, the results were updated in January and April 2023 due to delays in the clinical trial approval process. Data were extracted from the survey platform and reported using Excel (version 2310, Microsoft, Redmond, USA).

## Results

We collected information about all 28 partner sites that were involved in prospective patient recruitment in eight European countries within the framework of the STOPSTORM.eu consortium [7]. The following key information emerged from the survey: among the participating centres who were supposed to recruit patients prospectively, a total of 13 trials were designed and submitted to ethics committees (EC) for approval in seven of the eight countries involved in the STOPSTORM.eu consortium. The remaining centres that did not have an active clinical trial at this time recruited patients to undergo STAR through compassionate use. Detailed information on the clinical trials and the survey is provided in Table 1.

**Table 1:** List and brief description of all clinical trials for prospective data collection

#	Country	STOPSTORM partner site	Trial name	Registration no.	Start	Status	Finish	Planned no. of patients (recruited)	Study design, as approved by local EC	Legal basis of trial approval	EC concerns
C1	Czech Republic	FNO, NPO	Non-invasive ablation of ventricular tachycardia (NIRA-VT)	NCT03601832	01.07.2018	Closed (earlier than scheduled)	31.01.2020	10 (6 recruited)	Prospective single arm	Bail-out procedures, ethics committee FN Ostrava	No
C2	Czech Republic	FNO, NPO, IKEM	Stereotactic ablative radiosurgery of recurrent ventricular tachycardia in structural heart disease	NCT04612140	01.10.2020	Recruiting	01.05.2024	100 (20)	Randomized trial between STAR and catheter ablation	Modification of standard method, ethics committee FN Ostrava	No
G1	Germany	CAU/UKSH, UHEI/UMM, CHARITE	Radiosurgery for the treatment of refractory ventricular extrasystoles and tachycardias (RAVENTA) [9; 10]	NCT03867747	02.12.2019	Finished recruitment	30.10.2024	20	A prospective, single-arm, clinical interventional, feasibility trial (RAVENTA)	MDR Art. 82 Abs 1 formally MPG Art. 23b (exempt from medical device regulation)	No
CH1	Switzerland	USZ	Dose escalation for stereotactic cardiac radiation therapy of recurrent ventricular tachyarrhythmia—a single center, non-randomized phase II clinical trial	NCT05594368	01.10.2022	Recruiting	Unknown	15	Phase II according to ICH E8 § 3.1.3, risk category B according to ClinO, Art. 61. The treatment applied in the trial is authorized in Switzerland (not yet standard of care)	EC of the canton Zurich	No
P1	Poland	NRIO, GCM	Stereotactic management of arrhythmia—radiosurgery in treatment of ventricular tachycardia (SMART-VT)[5]	NCT04642963	11.09.2020	Completed	10.01.2023	11	Prospective single-arm study investigating the safety of non-invasive cardiac radiosurgery for the treatment of ventricular tachycardia (SMART-VT)	Ethical board decision based on the Act of 5 December 1996 on the professions of doctor and dentist with later amendments	No
P2	Poland	NRIO, GCM	Stereotactic management of arrhythmia—radiosurgery treatment and evaluation of response in ventricular tachycardia (SMARTER-VT)	NCT05913375	01.07.2023	Recruiting	01.05.2026	19	Prospective single-arm study investigating the effectiveness of non-invasive cardiac radiosurgery with a dose of 20 Gy for the treatment of ventricular tachycardia (SMARTER-VT)	Ethical board decision based on the Act of 5 December 1996 on the professions of doctor and dentist with later amendments	No
N1	Netherlands	AUMC	Stereotactic arrhythmia radiotherapy in the Netherlands no. 1 (STARNL-1); completed	NL7510	03. 01.2020	Completed	18.07.2022	6 (6 recruited)	Prospective pre–post intervention study	Ethical board (WMO-study [Dutch legislation])	No
N2	Netherlands	AUMC	Stereotactic arrhythmia radiotherapy in the Netherlands no. 2 (STARNL-2)	NCT05439031	15. 07.2022	Recruiting	01.06.2025	12	Prospective pre–post intervention study	Ethical board (WMO-study [Dutch legislation])	No
N3	Netherlands	UMCU	Prospective feasibility study of stereotactic arrhythmia radioablation (PRO-STAR)	NL9752	23.09.2021	Closed (earlier than scheduled)	01.10.2024	11 (3)	Prospective pre–post intervention study	Ethical board (WMO-study [Dutch legislation])	No
N4	Netherlands	MUMC, MAASTRO	Electroanatomic substrate-guided stereotactic ablative radiotherapy for refractory ventricular tachycardia (ELSTAR-VT)	NL9339	01.06.2021	Recruiting	Unknown	23	Prospective pre–post intervention study	Ethical board (WMO-study [Dutch legislation])	No
N5	Netherlands	LUMC	Mapping-guided stereotactic ablative radiotherapy (prospective study, not STOPSTORM harmonized, e.g. follow-up moments and questionnaires) [3]	NL7950	01.09.2019	Recruiting	Unknown	12	Prospective pre–post intervention study	Ethical board (WMO-study [Dutch legislation])	No
I1	Italy	UNITO, AUSL-RE, IRCCSDC, IRCCSOSM	Prospective cohort study for stereotactic arrhythmia radioablation (STAR) of refractory ventricular tachycardia —POPSTAR trial	NCT06294782	08.03.2022	Recruiting	31.12.2024	24	Observational registry and prospective clinical interventional trial (POP-STAR)	EC of Torino, EC of Verona-Rovigo, EC of Northern Emilia Romagna	Yes
S1	Spain	IMIM, SERMAS, FIHGUV	Radioablation non-invasive in ventricular tachycardia (RANIT)	[Not yet registered]	15.01.2020	Recruiting	2025	8	Observational registry and prospective clinical interventional trial	EC of Barcelona, EC of Madrid, EC of Valencia	No

### STOPSTORM protocol submission process and ethics approval

In general, the centres involved in STOPSTORM arranged to design and conduct prospective clinical trials at a national level under the lead of each respective principal investigator (PI). In most cases, the documents for each clinical trial were prepared and submitted for evaluation to the local EC by the PIs. Approval at the other centres then followed local regulations.

In most centres, the EC approval processes of the prospective protocols were performed without major obstacles, and the local ECs did not raise any critical issues or doubts.

In Italy, significant challenges were encountered in obtaining approval for the national clinical trial. One centre had the approval for sharing data of patients treated with STAR under compassionate use within STOPSTORM, but did not get approval for the interventional clinical trial (their proton therapy medical device holds an European Conformity[CE] label strictly for oncological use). At another Italian centre, the EC raised concerns about including STAR within the intended use of the local linear accelerator, interpreting STAR as an off-label treatment. Initially, the CE requested that the PI present the study as a clinical investigation of a medical device. However, after receiving clarifications from the clinical trial team, the EC acknowledged the explanations and approved the study as a prospective interventional trial under the CE label.

According to the survey results, no other ethical concerns or issues in the protocol submission processes were reported, either by the researchers or by the local ethics committees.

### Clinical trial description

Among the 31 partner sites in the STOPSTORM project, 28 centres were contributing to the registry through observational and/or prospective enrolment of patients at the time of the survey (since 2024, 19 additional partners have joined the project). A total of 13 clinical trials have been carried out or are currently ongoing at the original partner centres; detailed information on partners and clinical trials can be found in Table 1. The study designs were as follows: ten single-arm prospective mono-/multicentric trials (C1, G1, I1, N1‑5, P1, S1); one phase II dose escalation trial (CH1), one dose de-escalation trial (P2), and one randomized clinical trial comparing STAR to CA (C2). In some multicentre trials (C2, P1) patients are recruited and treated in two different centres, either due to the availability of medical devices and/or because the cardiology and radiation therapy departments are located in different facilities.

Not all studies complied with STOPSTORM guidelines for prospective trial development, including timing of follow-ups and administration of quality of life questionnaires. Eight studies were registered on the clinicaltrial.gov platform, while four of the five Dutch trials were registered in the Dutch national registry. The remaining clinical trial is not yet registered on clinicaltrial.gov. For a few studies, interim (G1, [9]) and final results (N5, [3]; P1, [5]) have already been published, along with the protocol for studies G1 and P1 [10].

**Fig. 1:**
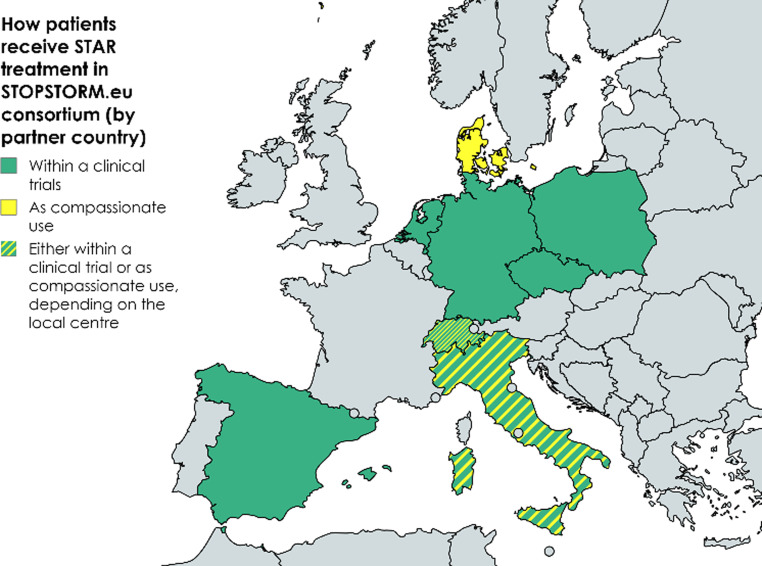
Overview of stereotactic arrhythmia radioablation (*STAR*) clinical trials or compassionate use among the initial Standardized Treatment and Outcome Platform for Stereotactic Therapy of Re-Entrant Tachycardia by a Multidisciplinary (STOPSTORM) Consortium partners

A minority of the STOPSTORM partners that intended to recruit patients do not have any active or planned trials. These are centres where STAR either cannot be performed or patients are treated through compassionate use; in the latter case, patients can sign a consent to be enrolled in the STOPSTORM registry and are still eligible for inclusion in the prospective cohort if their treatment and data meet the quality criteria. Figure 1 shows an overview of STAR clinical trials or compassionate use at STOPSTORM.eu consortium partners.

## Discussion

Our results show great variety in conceptual and scientific approaches among the clinical trials performed at STOPSTORM centres, with differences in experimental design, endpoints, planned follow-up, and the use of questionnaires investigating QoL. Even though the STOPSTORM.eu consortium has encouraged all partners recruiting patients prospectively to comply with the internal quality criteria and shared guidelines for data harmonization, the registry has an open character and will include data from all patients; thus, each country or partner could independently develop its own study protocol (or even continue to treat patients with compassionate use). Although respondents did not explicitly report major ethical concerns in the survey, the overall structure and implementation of the registry-based approach—including the heterogeneity of study designs and treatment protocols—may cause competing interests and differential quality levels of treatment delivery for STAR throughout Europe, factors that may have both clinical and ethical negative impacts. While data variability issues can be overcome through appropriate statistical analyses, a few ethical concerns related to autonomy and informed consent, privacy and sharing of data, differences in regulatory interpretations and evaluations among ECs, and access to last-resort experimental care must be addressed [8, 11, 12].

### Autonomy and informed consent

All prospectively treated patients, regardless of the cohorts they will be finally assigned to, signed consent to share their data in the STOPSTORM registry. The complexity of the informed consent process in STOPSTORM is amplified by the dual nature of participation: patients are first enrolled in local interventional studies on STAR (for which they sign a local informed consent specific to the experimental protocol) and then consent to share their data within the STOPSTORM registry. This dual consent process presents significant challenges. By agreeing to participate in both a local interventional study and a Europe-wide observational registry, patients may develop a misunderstanding of the nature and quality of the treatment they receive. Specifically, participation in a European project may lead to a therapeutic misconception, with patients incorrectly assuming that STAR treatment is standardized and its efficacy well established, simply because their data are included in a large-scale international registry. This misconception is particularly problematic because the STOPSTORM registry collects data from multiple interventional clinical trials conducted at different centres—each with potentially distinct scientific rationales, methodologies, and quality standards—and even from compassionate use treatments. To mitigate the risk of therapeutic misconception of patients falsely believing they are receiving standardized care of proven efficacy, it is essential to distinguish clearly between the two levels of participation: the local experimental study (with its own informed consent and ethical approval) and the European observational registry, which aggregates data from multiple heterogeneous sources. Therefore, it is crucial to clearly communicate the experimental nature of the treatment and the potential variability in treatment protocols across different centres in order to ensure that patients do not falsely believe they are receiving standardized care of proven efficacy.

This issue is particularly relevant given the vulnerability of refractory VT patients [13, 14]. Many of them are in a fragile health state or may even be hospitalized in intensive care units, which can compromise their decision-making capacity and/or their ability to select the treatment centre [14]. To safeguard the ethical principle of respect for patient autonomy, healthcare professionals have a responsibility to proactively empower patients to freely choose and to mitigate the risk of therapeutic misconception—not only by obtaining informed consent but also by ensuring that patients and their families fully understand the experimental nature of the treatment and the associated uncertainties [15].

### Privacy and data sharing

Given the amount of data sharing involved in STOPSTORM, concerns related to data governance and patient privacy exist. Ensuring consistent data security standards across multiple international centres is challenging, which is why the STOPSTORM.eu consortium emphasizes adherence to international data protection regulations, such as the General Data Protection Regulation (GDPR) in Europe, and promotes ethical data handling practices.

### Heterogeneity of protocol evaluations among ECs in Europe

Another significant ethical challenge in multicentric observational clinical trials like STOPSTORM concerns the variability in how local ECs interpret regulations and evaluate research protocols. In STOPSTORM, some ECs initially interpreted STAR as an off-label use of the linear accelerator, which led to delays in trial approval for several centres. According to those ECs, STAR fell outside the CE-labelled intended use of the device and was therefore considered a clinical investigation of a medical device under EU Regulation No. 2017/745. To address this issue, extensive discussions were held within the ERG and the NCs, and it was ultimately clarified that the focus of the trials was on the SBRT procedure itself, not on the device, which is CE-labelled for delivering SBRT to non-neoplastic pathologies, including cardiac indications [16, 17]. Consequently, the clinical trials were re-evaluated as investigations of a therapeutic procedure rather than a device and eventually approved. However, these regulatory challenges revealed a broader issue: while their independence allows ECs to evaluate research protocols free from external influence, safeguarding ethical standards and patient protection [18], it may also result in inconsistent interpretations of regulations across Europe [19]. As a result, the same protocol can receive different feedback or approval decisions from different ECs, leading to delays or discrepancies in patient recruitment and data collection [20–22].

The impact of these delays is twofold: (i) patients eligible for potentially life-saving or life-lengthening experimental treatments, such as STAR, may be excluded due to prolonged approval processes; (ii) the exclusion of these patients limits clinical research progress and reduces the amount of valuable data collected, particularly in rare conditions like refractory VT, where every patient counts [23]. While EU Regulation No. 536/2014 has harmonized clinical trial approval processes for pharmacological studies by allowing a single national evaluation valid across all centres in a member state [24], it does not apply to non-pharmaceutical research, including SBRT studies. Consequently, differences in EC interpretations continue to affect STOPSTORM and similar non-pharmaceutical multicentric trials [22; 25].

Until more uniform guidelines or binding regulations are established, it is essential that all centres involved in multicentric trials that fall outside EU Regulation No. 536/2014 collaborate closely from the protocol development stage, ensuring harmonized quality criteria to minimize the risk of divergent interpretations by ECs.

### Equity in access to experimental care

Differences in the experimental approaches and delays in trial approval processes across the STOPSTORM centres significantly impact equitable access to experimental therapies. This poses ethical concerns, particularly because STAR is a potentially life-saving treatment for refractory VT patients who have no other therapeutic options. However, despite STOPSTORM’s commitment to shared quality criteria for conducting prospective studies, the consortium cannot legally enforce uniform treatment standards across all centres. Patients at certain centres may receive higher-quality experimental care compared to others, raising ethical issues related to fair access to innovative therapies. This challenge highlights the need for better coordination among partners in multicentric trials to ensure consistent standards of care for patients, particularly for experimental treatments lacking standardized protocols. Jointly drafting study documents helps to clarify protocol concepts and key documents, such as informed consent, thus minimizing doubts and concerns from EC members.

Despite these challenges, the open registry approach also made it possible to overcome and manage some of the main hurdles to conducting clinical trials on STAR.

One of the main challenges is the limited number of eligible patients. Since recurrent VT is a rare medical condition, most STAR trials have small sample sizes, which may mean that collected data are insufficient to draw significant conclusions. The limited number of patients also increases the risk of bias and reduces the generalizability of results, making the development of clinical guidelines more challenging. By conducting numerous smaller trials at local levels and consolidating the data through the registry, the project effectively pools resources, increasing the volume of data collected and allowing the inclusion of diverse patient populations across different geographical and clinical settings.

Another barrier is the financial constraint. Organizing large multicentric clinical trials can be overly expensive due to complex and comprehensive regulatory requirements, including auditing and monitoring. These requirements, although essential for patient safety and research credibility, add a substantial burden at both the administrative and the financial level; this applies especially to non-profit research, which has typically limited funding options. Having a registry minimizes duplicative regulatory processes, such as repeated auditing and monitoring, which are usually required in multicentric interventional trials; reduces administrative burdens; and accelerates the research timeline. This allows for faster transitions from clinical investigation to implementation. Additionally, the reduction in administrative and financial burdens allows centres with limited research infrastructure to participate in high-impact clinical research.

### Limitations

This study has several limitations. Despite the aforementioned advantages of the consortium approach, the project might not provide data of the same quality as a protocol-driven multicentre prospective clinical trial would. Moreover, this study only takes into consideration centres within STOPSTORM, while STAR is under investigation in many more centres, both in and outside Europe. Many researchers involved in STOPSTORM have minor yet relevant conflicts of interest due to participation in the STOPSTORM.eu consortium and the national trials. Additionally, those questioned were involved in the process of developing the questionnaire, which might introduce bias. Lastly, an in-depth examination of national regulations and legislation on the approval of non-pharmacological clinical trials is lacking; although this is an important ethical issue, it requires specific legal expertise and goes beyond the scope of this ethical overview.

## Conclusion

This study provides a comprehensive analysis of the ethical, regulatory, and organizational issues faced by multicentric non-pharmaceutical clinical trials on a non-standardized treatment like STAR. Particular attention was paid to issues related to patient autonomy, therapeutic misconception, data governance, and equitable access to experimental care. We showed how the open registry approach adopted by STOPSTORM can be an effective strategy to overcome some of those barriers, such as limited patient eligibility and financial constraints. However, parties conducting similar clinical trials should be aware of all potential ethical concerns, especially the barriers to patient autonomy and to equitable access to experimental care. The European Union has taken many steps towards harmonizing clinical research processes and improving efficiency and transparency, but similar efforts still need to be made for non-pharmaceutical research. Collaborative and coherent engagement from all stakeholders—particularly researchers and regulatory bodies—is still necessary to harmonize, address potential inequities, and improve data quality.

The STOPSTORM.eu consortium is pivotal in establishing the role of STAR in refractory VT treatment, a milestone towards the development of a unified protocol for its clinical application, but the challenge of its optimization in a clinical setting needs extensive ethical oversight, regulatory standardization, and international cooperation.

## Supplementary Information

The three different versions of the survey

## Abbreviations

CA: Catheter ablation
EC: Ethics committee
ERG: Ethics & Regulations Working Group
GDPR: General Data Protection Regulation
ICD: Implantable cardioverter defibrillator
NC: National coordinators
QoL: Quality of life
SBRT: Stereotactic body radiotherapy
STAR: Stereotactic arrhythmia radioablation
STOPSTORM Consortium: Standardized Treatment and Outcome Platform for Stereotactic Therapy of Re-Entrant Tachycardia by a Multidisciplinary Consortium
VT: Ventricular tachycardia

## Footnotes

1 The group includes bioethics researchers, the project coordinator, and other advisory researchers, all of whom are involved in the STOPSTORM project.
