# Supplementary material for: Ethical considerations of prospective data collection for stereotactic arrhythmia radioablation (STAR): an overview from the STOPSTORM.eu consortium
Source: Strahlenther Onkol. 2025 Oct 29;202(7):734–43. doi: 10.1007/s00066-025-02480-2 (PMC13290918; doi:10.1007/s00066-025-02480-2)
Supplement: Supplementary file 1 — The three different versions of the survey [file 66_2025_2480_MOESM1_ESM.docx]

# **Appendix 1: the three different versions of the survey**

| **1° version, March 2022 (*close-ended questions replies*)** | | | |
| --- | --- | --- | --- |
| 1. How did already treated patients access treatment? *(With compassionate use/Through a local trial)* | | |  |
| 2. On what legal basis was the trial approved? | | |  |
| 3. What kind of trial has been approved? | | |  |
| 4. Was it a clinical investigation under EU Reg. 2017/45? | | |  |
| 5. How was the process of Ethics Committee (EC) submission in the approving of STAR? Were there difficulties? | | |  |
| 6. Is it possible to share EC approval documents? *(Yes/No)* | | |  |
|  | | | |
| **2° version, June 2022 - added/modified questions are underlined** | | | |
| 1. Have you already treated patients with STAR? (*Yes/no*) | | |  |
| 2. How did already treated patients access treatment? *(With compassionate use/Through a local trial)* | | |  |
| 3. Have you submitted a trial to enrol patients or plan to do so? (Yes/No/Planning to submit a trial protocol OR currently in the process of submitting a protocol) | | |  |
| 4.a What kind of protocol do you plan to submit? | 4.b What kind of protocol has been approved? |  |  |
| 5.a According to which regulations was the protocol written? | 5.b According to which regulations was the protocol approved? |  |  |
| 6. Was it a clinical investigation under EU Reg. 2017/45? | | |  |
| 7. How was the process of Ethics Committee (EC) submission in the approving of STAR? | | |  |
| 8. Did your local EC raise any concern? | | |  |
| 9. Did you encounter any difficulties? | | |  |
| 10. Is it possible to share EC approval documents (if and when available)? *(Yes/No)* | | |  |
|  | | | |
| **3° version, October 2022 - added/modified questions are underlined** | | | |
| 1. Have you already treated patients with STAR? *(Yes/no)* | | |  |
| 2. How did already treated patients access treatment? *(With compassionate use/Through a local trial)* | | |  |
| 3. Have you submitted a trial to enrol patients or plan to do so? *(Yes/No/Planning to submit a trial protocol/currently in the process of submitting a protocol)* | | |  |
| 4.a What kind of protocol do you plan to submit? | 4.b What kind of protocol has been approved? |  |  |
| 5.a According to which regulations was the protocol written? | 5.b According to which regulations was the protocol approved? |  |  |
| 6. Was it a clinical investigation under EU Reg. 2017/45? *(Yes/No)* | | |  |
| 7.a Do you anticipate any difficulties related to the protocol and/or its approval by the EC? | 7.b Did you encounter any difficulties related to the protocol and/or its approval? |  |  |
|  | 8.b Did your local EC raise any concern? Which ones? |  |  |
| 9. Is it possible to share EC approval documents (if and when available)? *(Yes/No)* | | |  |
